# Prostatic tissue in 46XX congenital adrenal hyperplasia: Case report and literature review

**DOI:** 10.1002/ccr3.3868

**Published:** 2021-01-27

**Authors:** Hamza Elfekih, Asma Ben Abdelkrim, Hajer Marzouk, Ghada Saad, Ayoub Gasmi, Moez Gribaa, Hounaida Zaghouani, Yosra Hasni, Amel Maaroufi

**Affiliations:** ^1^ Department of Endocrinology and Diabetology Farhat‐Hached University Hospital Sousse Tunisia; ^2^ Department of Radiology Farhat‐Hached University Hospital Sousse Tunisia; ^3^ Department of Cytogenetic and Reproductive Biology Farhat‐Hached University Hospital Sousse Tunisia

**Keywords:** 21‐hydroxylase deficiency, adrenal gland neoplasms, congenital adrenal hyperplasia, I2G/R356W, prostate, uterus

## Abstract

The presence of prostatic tissue, in addition to uterus and adrenal tumors, is possible in 46XX patients with CAH. Lesions of these organs are usually benign. However, complications including prostate and adrenal cancer were also reported.

## Graphical abstract

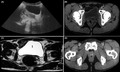

## 1 INTRODUCTION

Adrenal lesions are common in congenital adrenal hyperplasia. However, little is known about the dual presence of prostatic tissue and uterus in 46XX patients with this condition. Since such association is extremely rare, it is relevant for clinicians to be aware of, in order to detect complications including cancers early.

Congenital adrenal hyperplasia (CAH) is a group of enzymatic defects in adrenal steroidogenesis resulting in glucocorticoid insufficiency and excess of adrenal androgens.^1^ 21‐Hydroxylase deficiency (21‐OHD) is responsible for more than 90% of cases.^2^ It is caused by mutations in the *CYP21A2* gene, located on the short arm of chromosome 6. Although genotype‐phenotype correlation was well studied, it continues to display discordances.^3^ Herein, we describe the case of a patient with CAH due to 21‐OHD with male phenotype and female genotype presenting large adrenal mass, uterus, and prostatic tissue. We reviewed also the literature for similar cases with CAH, uterus, and prostate.

## 2 CASE REPORT

A 40‐year‐old man presented to the endocrinology department with a history of abdominal pressure and an ultrasound showing the presence of adrenal mass and uterus. The patient is the youngest child of a nonconsanguineous parents. His brother died at the age of 5 for an unknown cause. His sisters are married and have children. The patient, phenotypically male, was raised as a boy. There was no suspicion of genital ambiguity at birth. His past medical history was significant for precocious puberty at the age of 6 and evaluation for nonpalpable testes at the age of 15.

The patient identified himself as a male. On physical examination, he had a short stature (height = 152 cm), normal blood pressure, 15‐cm abdominal mass in the left upper quadrant, pubic hair Tanner score of 5, empty scrotum, hypospadias, and stretched penis size of 7 cm, and there was no gynecomastia. On biochemical examination, the patient had normal serum potassium, creatinine, and cortisol levels. Total testosterone was 9.1 ng/mL (normal range: 3‐12 ng/mL for males and 0.1‐0.7 ng/mL for females). 17‐Hydroxyprogesterone (17‐OHP), dehydroepiandrosterone sulfate (DHEAS), and androstenedione levels were elevated (Table 1).

**TABLE 1:** Biochemical parameters of the 46XX patient with congenital adrenal hyperplasia due to 21‐hydroxylase deficiency

Parameters	Value	Normal range
Fasting glycemia (mmol/L)	4.6	3.9‐6
Serum sodium (mmol/L)	139	136‐146
Serum potassium (mmol/L)	4.5	3.6‐4.6
Serum creatinine (µmol/L)	87	54‐115
17‐Hydroxyprogesterone (ng/mL)	247	0.5‐2.4
Dehydroepiandrosterone sulfate (ng/mL)	5213	1330‐4400
Androstenedione (ng/mL)	78	0.3‐3.1
Basal cortisol (ng/mL)	175	75‐220
Thyroid‐stimulating hormone (TSH) (mIU/L)	2.26	0.25‐4.5
Free thyroxine (FT4) (pg/mL)	10.8	7‐19
Follicle‐stimulating hormone (FSH) (mIU/L)	3.3	1.3‐11
Luteinizing hormone (LH) (mIU/L)	1.7	1.1‐10
Prolactin (ng/mL)	9.83	2‐10
Testosterone (ng/mL)	9.1	0.1‐0.7
Urinary sodium (mmol/day)	113	40‐220
Urinary potassium (mmol/day)	45	25‐125

**FIGURE 1:**
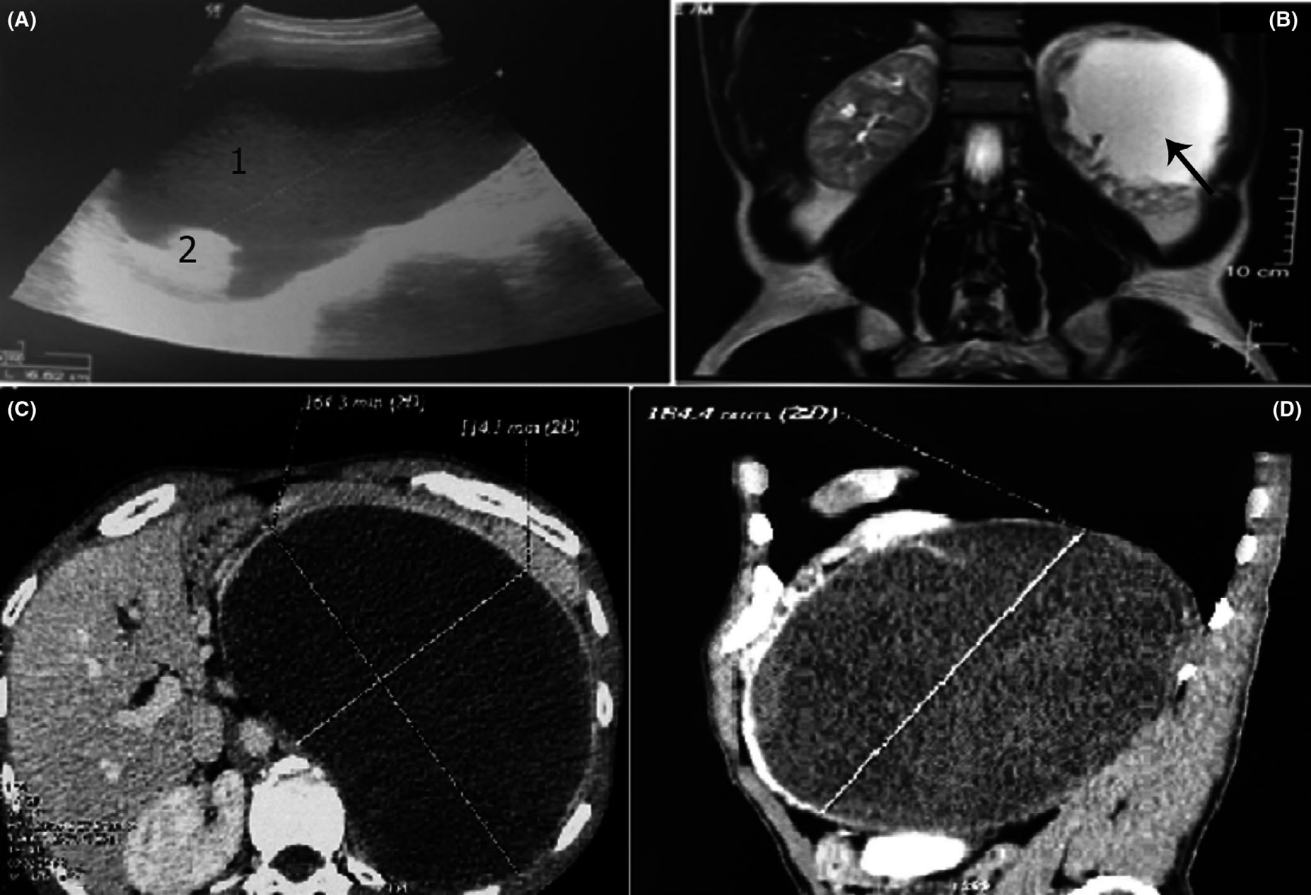
Abdominal imaging showing large adrenal mass in a 46XX patients with congenital adrenal hyperplasia due to 21‐hydroxylase deficiency. Abdominal ultrasound (A) showed left adrenal mass containing mixed cystic (1) and solid (2) components. The mass (arrow) was hyperintense on coronal T2‐weighted MRI (B). It measured 16.4 x 11.4 x 18.4 cm on abdominal CT scan (C‐D)

**FIGURE 2:**
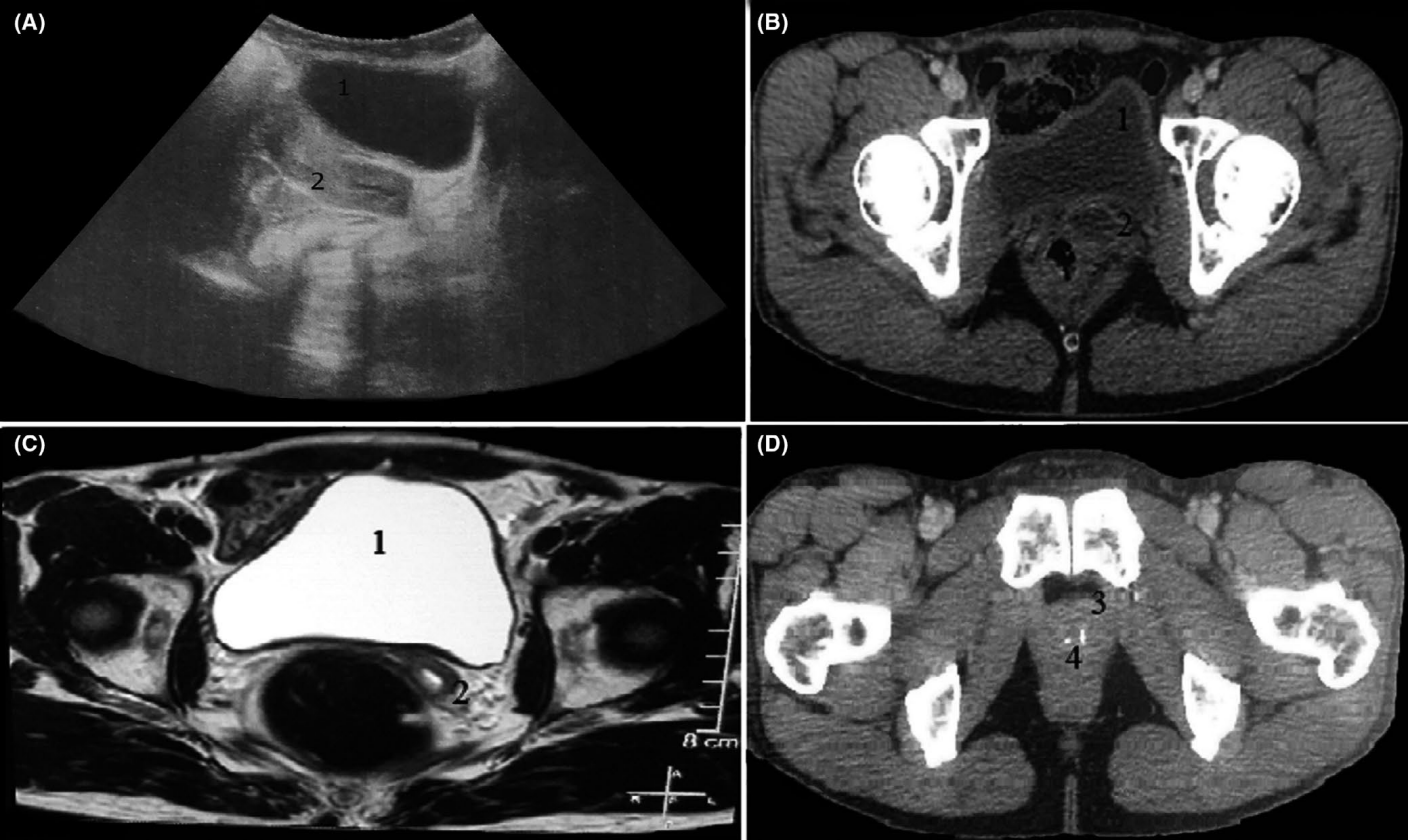
Pelvic imaging showing uterus and prostatic tissue in a 46XX patients with congenital adrenal hyperplasia due to 21‐hydroxylase deficiency. Urinary bladder (1) and uterus (2) can be seen on pelvic ultrasound (A), CT scan (B), and axial T2‐weighted MRI (C). Pelvic CT scan (D) showed also a prostatic tissue (3) containing two calcifications (4)

Ultrasonography, computed tomography scan (CT scan), and magnetic resonance imaging (MRI) of the abdomen and pelvis revealed left adrenal mass of 16.4 × 11.4 × 18.4 cm (Figure 1), right adrenal hyperplasia, uterine hypoplasia, and prostatic hypoplasia containing two calcifications (Figure 2). Gonads were not visualized. Genetic analysis was performed showing 46XX karyotype, using conventional R‐banding technique, and compound heterozygous mutation in the *CYP21A2* gene (I2G/R356W) based on Sanger sequencing method.

A diagnosis of classic CAH due to 21‐OHD was made, and the patient was proposed for hormonal treatment, unilateral adrenalectomy, hysterectomy, and gonadectomy (if found intraoperatively). We strongly suggested the need for performing prostate‐specific antigen (PSA) dosage and prostate biopsy since prostate cancers were described in these patients, as well as the necessity to screen parents and his sisters for mutation detection.^4^, ^5^ Female patients with CAH having prostatic tissue found in the literature are described in Table 2.^6^, ^7^, ^8^, ^9^, ^10^, ^11^, ^12^, ^13^, ^14^, ^15^, ^16^, ^17^ To the best of our knowledge, this is the 20th reported case.

**TABLE 2:** Female patients reported in the literature with congenital adrenal hyperplasia having prostatic tissue

Reference /Year	Clinical presentation	Biochemical /genetic analysis	Imaging finding	Prostate biopsy /surgery	Management
Crecchio [1865] ^6^	43‐year‐old phenotypically male patient with short stature, hypospadias, internal female anatomy, enlarged adrenals, and prostate	‐	‐	(Autopsy)	(Died in an apparent Addisonian crisis)
Schuhmann [1969] ^7^	Newborn with nonpalpable testicles, hypospadias, adrenal hyperplasia, uterus, ovaries, and prostate	‐	‐	(Autopsy)	(Volvulus surgery due to common mesenterium, infant died at the age of 3 months)
Kiviat et al [1978] ^8^	17‐year‐old phenotypically male with short stature, ambiguous genitalia, and palpable prostate	‐ 17‐OHP = 115 ng/mL Basal cortisol = 57 ng/mL ‐ 46, XX	Retrograde urethrography, cystourethroscopy: vagina, uterus, fallopian tubes	Biopsy	‐ Hysterosalpingo‐oophorectomy, vaginectomy, and insertion of prosthetic testicles ‐ Hydrocortisone and testosterone replacement
Heyns et al [1987] ^9^	60‐year‐old female with acute retention of urine, lower abdominal mass, short stature, and virilization signs	‐ 17‐OHP = 40.31 ng/mL Total testosterone = 2.59 ng/mL Basal cortisol = 14.31 ng/mL ‐ 46, XX	Cystoscopy: common urogenital sinus and appearance of a small middle lobe of prostatic tissue	Biopsy	‐ Total hysterectomy (multiple leiomyomas) with bilateral salpingo‐oophorectomy ‐ Transurethral resection of benign prostatic hyperplasia
Winters et al [1996] ^4^	32‐year‐old female with ambiguous genitalia	‐ 17‐OHP = 12 ng/mL Total testosterone = 2.63 ng/mL PSA = 13 ng/mL ‐ 46, XX	‐ Voiding cystourethrogram: urogenital sinus and uterus ‐ Bone scan: bone metastases of the prostate adenocarcinoma	Biopsy	‐ Bilateral salpingo‐oophorectomy and hypospadias repair ‐ External beam radiation therapy for adenocarcinoma of the prostate and for vertebral metastatic sites ‐ Hysterectomy for clear cell carcinoma of the endometrium ‐ Hydrocortisone and fludrocortisone
Kim et al [2004] ^10^	23‐year‐old woman with virilization signs and primary amenorrhea	‐ 17‐OHP = 130 ng/mL Total testosterone = 5 ng/mL Basal cortisol = 52 ng/mL ‐ 46, XX and absent *SRY* gene	Ultrasound, MRI: ovaries, fallopian tubes, uterus, and normal adrenal glands	Surgery	Total hysterectomy (ectopic prostatic tissue found in the cervix), bilateral salpingo‐oophorectomy, and reconstructive urethroplasty
Klessen et al [2005] ^11^	21‐year‐old woman with ambiguous genitalia and prolonged menstrual bleedings	‐ NS ‐ 46, XX	MRI: Left adrenal hyperplasia, uterus, ovaries, common urogenital duct, prostate‐like tissue	No	Genitoplastic correction of clitoral hypertrophy
Subramanian et al [2006] ^12^	14‐year‐old male with ambiguous genitalia	‐ Elevated urinary 17‐ketosteroids ‐ 46, XX	Genitography, ultrasound, MRI: adrenal hyperplasia, common urogenital sinus, hypoplastic uterus, ovaries, and prostate gland	No	Planned hysterosalpingo‐oophorectomy with vaginectomy and insertion of prosthetic testicles
Yeşilkaya et al [2008] ^13^	5‐year‐old male with precocious puberty and complete masculinization of the genitalia	‐ 17‐OHP = 35 ng/mL Basal cortisol = 30 ng/mL PSA = 0.29 ng/mL ‐ 46, XX	Pelvic ultrasound, MRI: adrenal hyperplasia, uterus, ovaries, and prostate	No	‐ Hydrocortisone supplementation ‐ Planned hysterosalpingo‐oophorectomy and insertion of prosthetic testicles
Paulino et al [2009] ^14^	5 girls with CAH	‐ PSA > 0.1 ng/mL ‐ 46, XX	MRI: prostate tissue	NS	NS
Lázaro et al [2013] ^15^	No	‐ 17‐OHP = 53.7 ng/mL Basal cortisol = 21.8 ng/mL ‐ 46, XX; absent *SRY*, *TSPY* and *DYZ3* gene; compound heterozygosis (I2G/R408C)	MRI: uterus, prostate, and nodules on the iliac chains	No	‐ Hysterectomy, bilateral adnexectomy, and resection of Leydig cell tumor ‐ 5 mg of prednisone per day
Yeşilkaya et al [2008] ^13^	5‐year‐old male with precocious puberty and complete masculinization of the genitalia	‐ 17‐OHP = 35 ng/mL Basal cortisol = 30 ng/mL PSA = 0.29 ng/mL ‐ 46, XX	Pelvic ultrasound, MRI: adrenal hyperplasia, uterus, ovaries, and prostate	No	‐ Hydrocortisone supplementation ‐ Planned hysterosalpingo‐oophorectomy and insertion of prosthetic testicles
Paulino et al [2009] ^14^	5 girls with CAH	‐ PSA > 0.1 ng/mL ‐ 46, XX	MRI: prostate tissue	NS	NS
Lázaro et al [2013] ^15^	10‐year‐old phenotypically male with short stature, bilateral cryptorchidism, and accelerated sexual development	‐ 17‐OHP = 53.7 ng/mL Basal cortisol = 21.8 ng/mL ‐ 46, XX; absent *SRY*, *TSPY* and *DYZ3* gene; compound heterozygosis (I2G/R408C)	MRI: uterus, prostate, and nodules on the iliac chains	No	‐ Hysterectomy, bilateral adnexectomy, and resection of Leydig cell tumor ‐ 5 mg of prednisone per day
Fang et al [2013] ^16^	64‐year‐old man with short stature, ambiguous genitalia	‐ 17‐OHP > 247.8 ng/mL Total testosterone = 9.51 ng/mL Normal ACTH and cortisol levels PSA = 3.6 ng/mL ‐ 46, XX	Ultrasound, CT scan: large right adrenal mass, bilateral diffuse adrenal enlargement, uterus, ovaries, prostate tissue containing a speck of calcification	No	‐ Corrective surgery of hypospadias ‐ Right adrenalectomy, hysterectomy, and bilateral salpingo‐oophorectomy ‐ No regular corticosteroid replacement
Teixeira et al [2014] ^17^	Neonate with nonpalpable testicles	‐ NS ‐ 46, XX	MRI: adrenal hyperplasia, uterus, hydrocolpos, ovaries, prostate tissue	No	Planned feminizing surgery
Wesselius et al [2017] ^5^	64‐year‐old phenotypically male with CAH and complete virilization	‐ PSA = 4.6 ng/mL ‐ 46, XX	MRI: rudimentary prostate	Biopsy	‐ Hysterectomy, bilateral adnexectomy, dexamethasone, and testosterone replacement ‐ Low anterior resection of sigmoid adenocarcinoma ‐ External beam radiotherapy for prostate carcinoma
Current case	40‐year‐old phenotypically male with precocious puberty, short stature, and ambiguous genitalia	‐ 17‐OHP = 247 ng/mL Total testosterone = 9.1 ng/mL ‐ 46, XX and compound heterozygosis (I2G/R356W)	Ultrasound, CT scan, MRI: adrenal hyperplasia, large left adrenal mass, uterus, and prostatic tissue containing two calcifications	No	Planned hormonal substitution, hysterectomy, gonadectomy, left adrenalectomy, dosage of PSA, and transrectal biopsy of the prostate

Note

Abbreviations: 17‐OHP, 17‐hydroxyprogesterone; CAH, congenital adrenal hyperplasia; MRI, magnetic resonance imaging; NS, not specified; PSA, prostate‐specific antigen.

## 3 DISCUSSION

21‐OHD is the most common cause of 46XX disorder of sex development. It is responsible for androgens excess, resulting in virilization and ambiguous genitalia. Internal genitalia are usually normal in these patients.^13^

The presence of prostate tissue in 46XX patients with CAH has been described in a limited number of cases (Table 2). In the study of Paulino et al, its incidence in the classical form of CAH was 15.6%.^14^ The presence of prostate tissue was related to the level of adrenal androgens and the timing during fetal development.^12^, ^13^ In females, the paraurethral gland or Skene gland is homologous to the prostate gland in males. In the presence of androgen excess, this gland may develop into identical male prostate tissue.^5^ Patients with Prader type III, IV, and V external genitalia or with excessive adrenal androgens stimulation before the 16th week of fetal development may develop a prostatic tissue.^12^, ^13^

Our patient had Prader V external genitalia, which could explain the presence of the prostate gland. Calcifications found inside the prostatic tissue in our case were reported only once in a 64‐year‐old phenotypic male with 46XX karyotype.^16^ PSA dosage and transrectal biopsy of the prostate are useful to detect adenocarcinoma. This malignant prostate tumor was described twice in 46XX patients with CAH due to 21‐OHD.^4^, ^5^ Benign prostatic hyperplasia was described only once.^9^ Additionally, PSA dosage could be useful to detect prostatic tissue in patients with CAH. A cutoff level of 0.1 ng/mL showed 100% sensitivity and 88.9% specificity.^14^

Adrenal masses are common in CAH, up to 82% in homozygous and 45% in heterozygous patients.^16^ The size of both adrenal glands and tumors has been described to be significantly correlated with a poor hormonal control status, and it can regress with adequate treatment.^17^ In our case, the long evolution of the disease without medications controlling hormonal parameters could explain the large size of the adrenal mass.

A recent meta‐analysis confirmed the high prevalence of adrenal tumors (23.6%) in patients with 21‐OHD, which are most likely to be benign, particularly myelolipoma with a prevalence of 8.6% in those patients.^18^ Additionally, two cases of pheochromocytoma and five cases of adrenal cortical cancer in patients with CAH were also reported in the literature.^18^ One of the cases of adrenocortical carcinoma was associated with giant bilateral myelolipomas.^19^ No cystic soft‐tissue tumor of the adrenal gland was reported in patients with 21‐OHD to the best of our knowledge. Moreover, it is known that large adenomas may show not only cystic components but also calcifications and hemorrhagic areas.^20^ Our patient requires a histopathological examination given the large size and the dual component of the tumor, in order to rule out a malignant adrenal mass.

Serum androgens levels were high in our case. In the study of Lee et al, 79% of patients with 21‐OHD had serum DHEAS levels above the normal range, without significant difference between salt wasting (SW) and simple virilizing (SV) forms, and all patients had serum androstenedione and testosterone levels above the normal range.^21^ Note that even benign lesions can have rarely an increased concentration of these hormones (3%) in patients without CAH.^22^

CAH is a heterogeneous group of enzymatic defects with classic and nonclassic types. The SW phenotype is the most severe and common form.^16^ Although phenotype‐genotype correlation was well studied, variability in phenotype has been described with several mutations.^3^

In our case, the patient had compound heterozygous mutation (I2G/R356W). R356W is a missense mutation in exon 8, while I2G is a splice site mutation in intron 2. The R356W mutation was associated usually with SW form, and the I2G mutation was associated with both SW and SV forms.^3^, ^23^ Our patient had SV phenotype despite the presence of two mutations associated with SW. It is well known in 21‐OHD patients with compound heterozygous mutation that the clinical phenotype is correlated with the mutation retaining the most enzyme activity, which is expressed by the less severely mutated allele. In our case, the I2G mutation was responsible for the SV phenotype.^24^ However, in a large cohort of 1,507 cases with CAH, only 4 patients had the SV form from 22 with the I2G/R356W mutation.^23^ This fact highlights the great phenotypic variability, which is modulated by the level of residual enzymatic activity. In a Tunisian study made by Kharrat et al, compound heterozygous mutations were found mainly in nonconsanguineous patients, which could indicate a high prevalence and diversity of mutations in the Tunisian population.^25^

In the cases cited in table 2, only one mutation was specified, which was also a compound heterozygosis (I2G/R408C). Further studies are required to determine whether the presence of prostate is prevalent or not with some specific mutations.

To our knowledge, this is the first case reporting the presence of prostate in 46XX patient and the identification of I2G/R356W mutation in a Tunisian patient. The most frequent mutation in classical forms of 21‐OHD in Tunisian population was Q318X, with 26%‐35.3%. I2G and R356W mutations were present in 6%‐17.6% and 1%‐2.9% of cases, respectively. This distribution of mutation frequencies in Tunisian patients was reported to be different from other countries.^25^, ^26^

In conclusion, we described a rare case of prostatic tissue in 46XX Tunisian patient with CAH due to 21‐OHD, in addition to reporting similar cases found in the literature. The presence of a prostate gland in females with CAH is well proven. Its frequency may be underestimated due in part to the lack of systematic screening but also to unawareness of physicians of the possible presence of prostatic tissue in those patients.

## CONFLICT OF INTEREST

None declared.

## AUTHOR CONTRIBUTIONS

Hamza ELFEKIH: involved in conception and design of study, literature search, and drafting of article. Asma BEN ABDELKRIM, Hajer MARZOUK, and Ghada SAAD: involved in design of manuscript, literature search, and drafting of article. Ayoub GASMI and Hounaida ZAGHOUANI: involved in radiographic image acquisition and interpretation. Moez GRIBAA: involved in genetic testing. Yosra HASNI and Amel MAAROUFI: involved in design of manuscript, drafting of article, and final approval.

## ETHICAL APPROVAL

The patient consented to the reporting of this case in a scientific publication.

## Data Availability

The data of this case are available from the corresponding author upon reasonable request.
